# EBV Tegument Protein BNRF1 Disrupts DAXX-ATRX to Activate Viral Early Gene Transcription

**DOI:** 10.1371/journal.ppat.1002376

**Published:** 2011-11-10

**Authors:** Kevin Tsai, Nadezhda Thikmyanova, Jason A. Wojcechowskyj, Henri-Jacques Delecluse, Paul M. Lieberman

**Affiliations:** 1 The Wistar Institute, Philadelphia, Pennsylvania, United States of America; 2 Cell and Molecular Biology Program, The University of Pennsylvania, School of Medicine, Philadelphia, Pennsylvania, United States of America; 3 German Cancer Research Center, ATV-F100, Heidelberg, Germany; University of Wisconsin-Madison, United States of America

## Abstract

Productive infection by herpesviruses involve the disabling of host-cell intrinsic defenses by viral encoded tegument proteins. Epstein-Barr Virus (EBV) typically establishes a non-productive, latent infection and it remains unclear how it confronts the host-cell intrinsic defenses that restrict viral gene expression. Here, we show that the EBV major tegument protein BNRF1 targets host-cell intrinsic defense proteins and promotes viral early gene activation. Specifically, we demonstrate that BNRF1 interacts with the host nuclear protein Daxx at PML nuclear bodies (PML-NBs) and disrupts the formation of the Daxx-ATRX chromatin remodeling complex. We mapped the Daxx interaction domain on BNRF1, and show that this domain is important for supporting EBV primary infection. Through reverse transcription PCR and infection assays, we show that BNRF1 supports viral gene expression upon early infection, and that this function is dependent on the Daxx-interaction domain. Lastly, we show that knockdown of Daxx and ATRX induces reactivation of EBV from latently infected lymphoblastoid cell lines (LCLs), suggesting that Daxx and ATRX play a role in the regulation of viral chromatin. Taken together, our data demonstrate an important role of BNRF1 in supporting EBV early infection by interacting with Daxx and ATRX; and suggest that tegument disruption of PML-NB-associated antiviral resistances is a universal requirement for herpesvirus infection in the nucleus.

## Author Summary

Persistent infection by Epstein-Barr virus (EBV) is associated with a variety of diseases, including lymphoid and epithelial tumors. Despite a wealth of information on the mechanism of viral persistence, relatively little is known about the early steps of EBV infection and viral gene activation. Host cells actively mount resistances against viral infection, which viruses need to overcome to invade the cell. We have found that among the proteins packaged in the EBV viral particle, BNRF1 plays an important role of counteracting cellular defenses. We show that EBV protein BNRF1 binds to the cellular protein Daxx and disassembles the Daxx-ATRX complex, where both Daxx and ATRX are cellular proteins known to inhibit viral gene expression. We also confirm that BNRF1 can promote expression of early viral genes, and that Daxx-binding by BNRF1 is required for this function. Finally, we demonstrate that Daxx and ATRX repress viral gene expression during latency. We conclude that BNRF1 disassembles cellular antiviral defense machinery to promote expression of viral genes in the host cell.

## Introduction

Epstein-Barr virus (EBV) is a member of the human gammaherpesvirus subfamily that infects over 90% of the global adult population [1], [2]. EBV preferentially establishes latent infection in B-lymphocytes but can also infect epithelial cells [3], [4]. EBV primary infection is one of the main causes of infectious mononucleosis (IM); while EBV latent infection is associated with multiple malignancies such as nasopharyngeal carcinoma, Burkitt's lymphoma, and Hodgkin's lymphoma [3], [4]. Furthermore, EBV is responsible for the majority of lymphoproliferative diseases associated with AIDS and immunosuppression following organ transplant [5]. Like all herpesviruses, EBV exists in a dynamic balance between productive and latent infection. The factors that regulate the fate decisions for lytic reactivation from latency have been investigated in some detail, but relatively little is known about the fate regulation during the earliest stages of primary infection.

Upon entry into the nuclear compartment, herpesvirus DNA genomes must confront several intrinsic anti-viral resistances that restrict viral gene expression and replication. One prominent nuclear structure involved in antiviral resistances is the PML nuclear body (PML-NB), also referred to as nuclear domain 10 (ND10). PML-NBs are nucleoplasmic protein aggregates mainly consisting of (but not limited to) the components PML, Sp100, Daxx, and ATRX [6], [7]. The size and abundance of PML-NB is interferon inducible [8], [9], [10], and over-expression of the PML protein represses viral infection [11]. PML-NB is the nuclear localization site of many DNA viruses, including Herpes Simplex virus (HSV-1), Human Cytomegalovirus (HCMV) and Adenovirus (Ad5) [12], [13]. These viruses then modify the morphology and/or protein composition of PML-NBs shortly after infection [12], [14]. The mechanism of PML-NB-mediated antiviral repression is not clearly determined. PML, Sp100, and Daxx are all associated with transcription repression, and this function may act on viral genomes [15]. Daxx can act as a transcription co-repressor of many cellular transcription factors [16], [17], [18], [19], and forms repressive transcription complexes with histone deacetylases (HDACs) [20], [21] and DNA methyltransferase I (DNMT I) [22], [23]. Daxx has been shown to induce heterochromatin markers on the HCMV genome and repress viral gene expression in a HDAC dependent manor [24], [25]. Daxx also forms a chromatin-remodeling complex with ATRX [26] and both can form a repression complex at heterochromatin [27]. Furthermore, RNA interference (RNAi) studies have shown that knockdown of Daxx or ATRX can result in a higher infection level of HCMV [28], [29], [30] and also relieve the infection defect of mutant HSV deficient in disrupting PML-NB [31].

Herpesviruses confront intrinsic anti-viral resistances immediately upon entering the host cell nucleus, and therefore must counteract these resistances at the earliest possible time points to initiate viral gene expression. Herpesvirus tegument proteins, which are pre-packaged and delivered with the infectious virion, are strategically positioned to counteract the intrinsic anti-viral defenses and support the early steps of infection [32]. Both alpha- and beta- herpesviruses encode tegument proteins that regulate early events during lytic replication, including the disruption of the PML-NBs. HSV-1 immediate early gene ICP0, disrupts PML-NB structure by degrading the core component PML [33], [34], [35] and eliminating SUMO-modified Sp100 [36]; while HCMV tegument protein pp71 displaces ATRX and subsequently degrades Daxx [24], [30]. Both ICP0-deficient HSV-1 and pp71-deficient HCMV mutants are deficient in infection, where viral gene expression is shutdown, resulting in a dormant viral genome [29], [37], [38]. Interestingly, it has been reported that disruption of PML-NB by ICP0 is mediated by *de novo* synthesized ICP0, instead of tegument delivered ICP0 protein, suggesting that this event is coordinated with early viral gene activation or, perhaps, reactivation from latent infection [35]. We have previously shown that EBV genomes localize to and then disrupt PML-NB during lytic replication; while latent EBV episomes are segregated away from PML-NBs during latency [39]. EBV regulatory proteins, including the lytic cycle immediate early gene Zta (also referred to as BZLF1, ZEBRA, and Z), and latency associated EBNA1 and EBNA-LP, have been implicated in PML-NB interactions [40], [41], [42]. However, it remains unclear if PML-NBs regulate early events associated with viral gene expression upon EBV nuclear entry, and if an EBV tegument protein modulates this intrinsic defense.

The EBV major tegument protein BNRF1 is one of the most abundant tegument proteins in the virion [43] and is essential for the establishment of viral latent infection [44], yet its function is largely unknown. BNRF1 homologues are present in all gammaherpesviruses but absent in the alpha- and beta- herpesvirus subfamilies. All BNRF1 orthologues share regions homologous to the cellular enzymes Phosphoribosylformylglycineamide Amidotransferase (FGARAT) and Aminoimidazole ribonucleotide (AIR) synthetase, ATP-dependent enzymes in the 4^th^ and 5^th^ steps of the purine *de novo* biosynthesis pathway. However, no enzymatic activity has been found in any BNRF1 orthologues. In a knockout study, transfected BNRF1-deficient EBV genomes can reactivate from latency, produce morphologically normal virions, and the progeny can enter cells with little observed defects [44]. Yet, upon infection of B cells the mutant virus showed a 20-fold lower expression of a viral latency associated gene EBNA2 and failed to induce B cell transformation [44]. This suggests an important role of BNRF1 in supporting early infection. Furthermore, the BNRF1 orthologue encoded by murine herpes virus 68 (MHV68), tegument protein ORF75c, induces PML degradation and is essential for initiation of viral gene expression [45], [46].

Here, we demonstrate that EBV BNRF1 is a novel PML-NB-interacting viral protein, and that this interaction is important for supporting EBV primary infection. We first show that Daxx is a primary cellular interaction partner of BNRF1. BNRF1 co-localizes with Daxx at PML-NB foci while disrupting the Daxx-ATRX complex. Furthermore, we identify a novel Daxx interaction domain on BNRF1. This domain is essential for BNRF1 to interact with Daxx, localize to PML-NB, and displace ATRX from Daxx. We then show that BNRF1 supports EBV primary infection and promotes the expression of viral genes soon after viral genomes enter the cell, and that the Daxx interaction domain contributes to these functions. Lastly, we show that knockdown of either Daxx or ATRX results in disruption of viral latency, suggesting that Daxx and ATRX play a role in the restriction of viral gene expression. Our study suggests that EBV tegument protein BNRF1 disassemble the Daxx-ATRX antiviral resistance complex to enable viral gene expression after cell invasion, and likely regulate the chromatin organization for the establishment of latent infection.

## Results

### BNRF1 interacts with cellular protein Daxx

**Figure 1:**
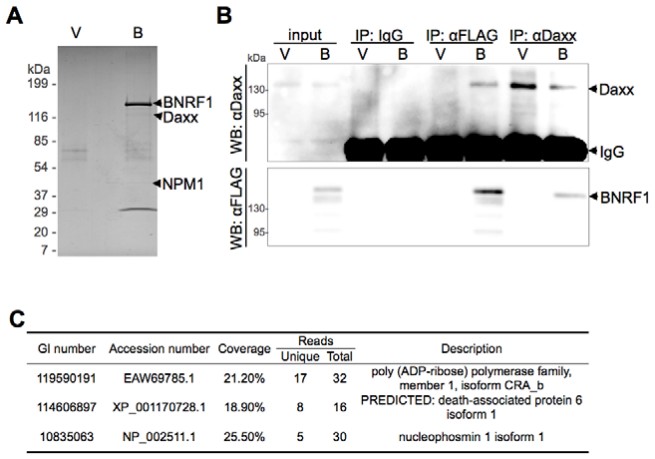
BNRF1 binds the cellular protein Daxx. (A) Colloidal blue stained SDS-PAGE of FLAG-immunoprecipitated BNRF1 and interacting partners. 293T cells were stably transfected with empty FLAG vector (V) or FLAG-tagged BNRF1 (B). Cell lysates were subject to Immunoprecipitation (IP) by anti-FLAG antibodies, then analyzed by SDS-PAGE. Bands unique to lane B were cut out and identified by LC/MS/MS. (B) IP confirmation of BNRF1/Daxx interaction. 293T cells were transiently transfected with empty vector (V) or wild-type BNRF1 (B). Cells harvested two days post-transfection were subject to IP with non-specific IgG, anti-FLAG or anti-Daxx antibodies, and analyzed by Western blot (WB) with anti-FLAG or anti-Daxx antibodies. (C) Summary of LC/MS/MS data from FLAG-BNRF1 purification. Genebank accession number (GI), percent of peptide coverage, number of peptides identified, and protein name are indicated.

To characterize the biological properties of the EBV major tegument protein BNRF1, we took a proteomic approach to screen for potential cellular interaction partners. BNRF1 was cloned into a 3x FLAG tag expression vector under the control of a CMV promoter. 293T cells were then stably transfected with either FLAG-vector or FLAG-tagged BNRF1. Nuclear extracts from stable cell lines were subject to immunopurification (IP) with a FLAG antibody, and then analyzed by SDS-PAGE (Fig. 1A). Bands unique to the BNRF1 lane (B) were cut out and analyzed by liquid chromatography-tandem mass spectrometry (LC/MS/MS). The major identified species was BNRF1, but substoichiometric proteins enriched in the BNRF1 IP were also identified, including Daxx, nucleophosmin (NPM1), and PARP1 (Fig. 1C). We subsequently confirmed in BNRF1 transiently transfected 293T cells that Daxx co-precipitates with BNRF1 (Fig. 1B) by both FLAG pull-down and the Daxx reverse pull-down, indicating a stable *in vivo* interaction between BNRF1 and Daxx. Neither PARP1 nor NPM1 interaction with BNRF1 could be validated by subsequent co-IPs (data not shown), we therefore focused our efforts on characterizing the interaction with Daxx.

### BNRF1 utilizes a novel Daxx interaction domain

**Figure 2 ppat-1002376-g002:**
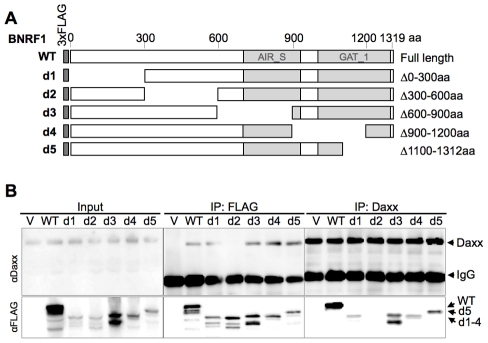
Mapping the Daxx-interaction domain on BNRF1, and the effect of BNRF1 on the Daxx-ATRX complex. (A) Diagram of wild-type BNRF1 (WT) and mutation constructs with 300 aa deletions (d1-d5). Dark gray block denotes the amino-terminal FLAG tag. Light gray blocks denote regions with sequence homology to the cellular enzymes Aminoimidazole ribonucleotide synthetase (AIR_S) and Type 1 glutamine amidotransferase (GATase1, an enzymatic domain of FGARAT), as identified by the National Center for Biotechnology Information (NCBI) conserved domain search. (B) IP pull down analysis of the BNRF1 deletion constructs. 293T cells were either transfected with empty FLAG vector (V), the BNRF1 constructs WT, or mutants d1-d5. Cell lysates of transfected cells were then subject to IP pull-downs with non-specific IgG, anti-FLAG, or anti-Daxx- antibodies, then Western blots were probed for Daxx (top panels), and FLAG-tagged proteins (lower panels). Input is shown for each mutant in the left most panels.

To further characterize the interaction between BNRF1 and Daxx, we introduced serial deletions on the FLAG-BNRF1 expression plasmid. We first made five deletion constructs of BNRF1, sequentially deleting regions coding for 300 amino acids (Fig. 2A, constructs d1 through d5). We then performed IPs with either control IgG, αFLAG, or αDaxx on lysates of cells transfected with the BNRF1 deletion constructs. Daxx co-precipitated in the FLAG IP for all of the BNRF1 mutants with the exception of the BNRF1 300–600 aa deletion mutant (d2) (Fig. 2B, middle panels). Similarly, all of the FLAG-BNRF1 mutants, with the exception of d2, co-precipitated with Daxx IP (Fig. 2B, right panels). Since d2 was expressed and recovered by FLAG IP to similar levels as other BNRF1 mutants capable of interacting with Daxx, we conclude that a putative Daxx-interaction domain is located in the region between 300-600aa of BNRF1.

**Figure 3 ppat-1002376-g003:**
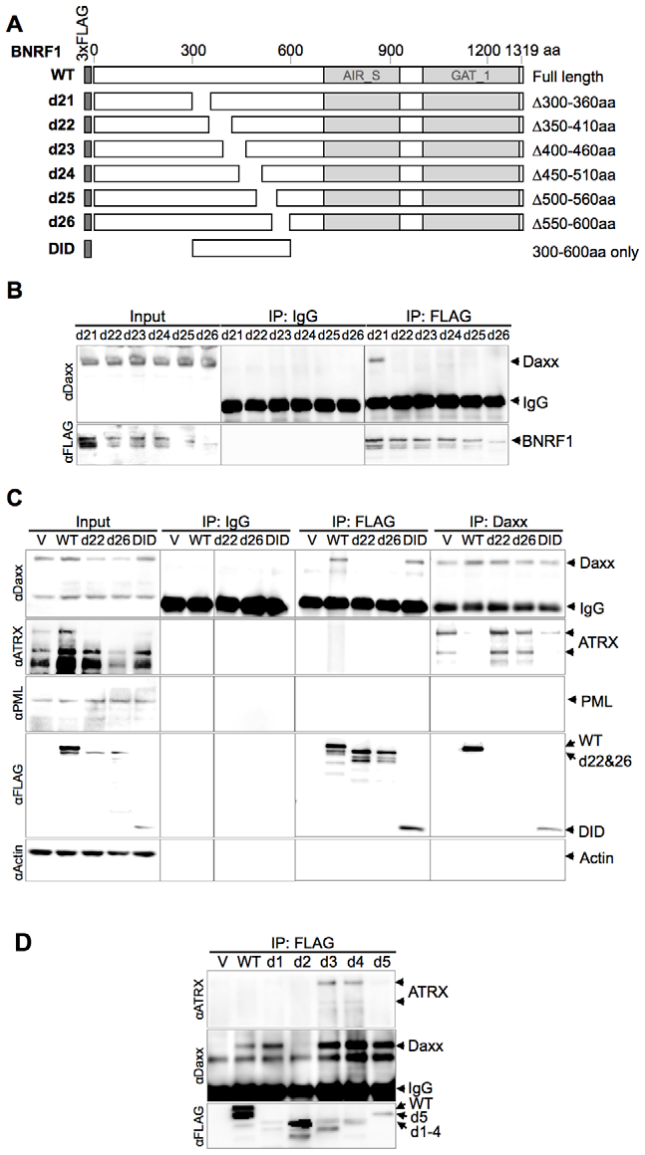
The Daxx-interaction domain on BNRF1 is located between sites 360-600 aa, and BNRF1 disrupts Daxx-ATRX binding. (A) Diagram of BNRF1 mutation constructs with 60 aa deletions (d21–d25) within the 360–600 aa Daxx-interaction domain (DID) and the DID only. Blocks in the diagram drown as Fig. 2A. (B) IP analysis of BNRF1 60 aa deletion constructs. 293T cells were transfected with various BNRF1 expression vector constructs, and cell lysates were subject to IP pull downs with non-specific IgG and anti-FLAG antibodies, then Western blots were probed for Daxx (top panel), or FLAG-tagged proteins (lower panel). (C) IP analysis of 293T cells transfected with vector control (V), WT BNRF1 (WT), BNRF1 deletion mutants d22, d26, or DID. Input, IgG control IP, FLAG IP, or Daxx IP (panels left to right) as indicated above each panel. Western blots of IPs were probed with antibody to Daxx, ATRX, PML, FLAG, or Actin, as indicated to the left of each panel. (D) IP Western of 293T cells transfected with vector control (V), WT-BNRF1 (WT), or BNRF1 deletion mutants d1, d2, d3, d4, or d5. FLAG-IPs were analyzed by Western blot with antibodies to ATRX (top panel) Daxx (middle panel), or FLAG (lower panel).

We then further made six serial deletions of 60 amino acids in the 300–600 aa region (Fig. 3A, constructs d21 through d26) to narrow down the suspected Daxx-interaction domain to a smaller region. After a subsequent round of IP pull-downs, we found that all BNRF1 deletions, with the exception of d21, were defective in binding Daxx (Fig. 3B), suggesting that the 360–600 aa region of BNRF1 is responsible for interaction with Daxx. To determine if this region was sufficient for interaction with Daxx, we expressed only the 300–600 aa region in the FLAG-expression vector (Fig. 3A, construct DID) and performed IP pull-downs. We found that this region bound Daxx as efficiently as WT-BNRF1, in both FLAG IP and in the reverse IP with anti-Daxx antibody (Fig. 3C). Notably, we failed to find sequence homology of this Daxx interaction domain with any known protein motif, and this domain is also distinct from the FGARAT and AIR synthetase homology regions. These findings suggests that BNRF1 utilizes a previously unknown motif to bind Daxx, and that the Daxx interaction domain (300–600 aa) may contain a complex protein fold sensitive to smaller truncation deletions.

### BNRF1 disrupts the Daxx-ATRX chromatin remodeling complex

Daxx forms a chromatin remodeling complex with ATRX [26] and ATRX has been implicated in the transcriptional repression of both HSV-1 and HCMV during the early steps of infection [30]. Moreover, both HSV-1 and HCMV utilize viral encoded proteins that disrupt the interaction between Daxx and ATRX [30], [31]. To determine if BNRF1 also disrupted the interaction between Daxx and ATRX, we assayed the effect of WT and mutant BNRF1 proteins on the co-IP of Daxx with ATRX. We observed that WT BNRF1 disrupted the interaction between Daxx and ATRX (Fig. 3C, 2^nd^ panel from top, right). However, deletion mutants d22 and d26, which fail to interact with Daxx, did not disrupt ATRX binding in Daxx IP assays (Fig. 3C, 2^nd^ panel from top, right). Interestingly, the Daxx interaction domain by itself (DID), which binds Daxx efficiently, could only partially disrupt ATRX binding. This suggests that Daxx binding by BNRF1 is necessary, but not sufficient for the disruption of ATRX with Daxx. We also found no evidence that BNRF1 co-IPs with PML (Fig. 3C, 3^rd^ panel from top).

To determine whether any other domains of BNRF1 contribute to the disruption of ATRX from Daxx, we assayed FLAG-BNRF1 IPs for ATRX binding using the set of larger BNRF1 deletions examined in Figure 2 (Fig. 3D). We found that WT BNRF1 did not co-IP with ATRX, although it efficiently pulled down Daxx. The BNRF1 d2 mutant failed to pull down Daxx or ATRX, as expected. In contrast, the BNRF1 d3 and d4 mutants, which disrupts most of the FGARAT and AIR synthetase homology regions, efficiently pulled down both ATRX and Daxx. The d1 and d5 truncations, which lie outside of the FGARAT and AIR synthase homology regions, pulled down only Daxx but not ATRX, suggesting it efficiently disrupted the ATRX-Daxx interaction similar to WT. These data suggest that the FGARAT and AIR synthetase homology regions of BNRF1 may contribute to the disruption of ATRX-Daxx complex.

### BNRF1 co-localizes with Daxx to PML nuclear bodies and disperses ATRX from nuclear bodies, in a Daxx interaction domain-dependent manner

**Figure 4 ppat-1002376-g004:**
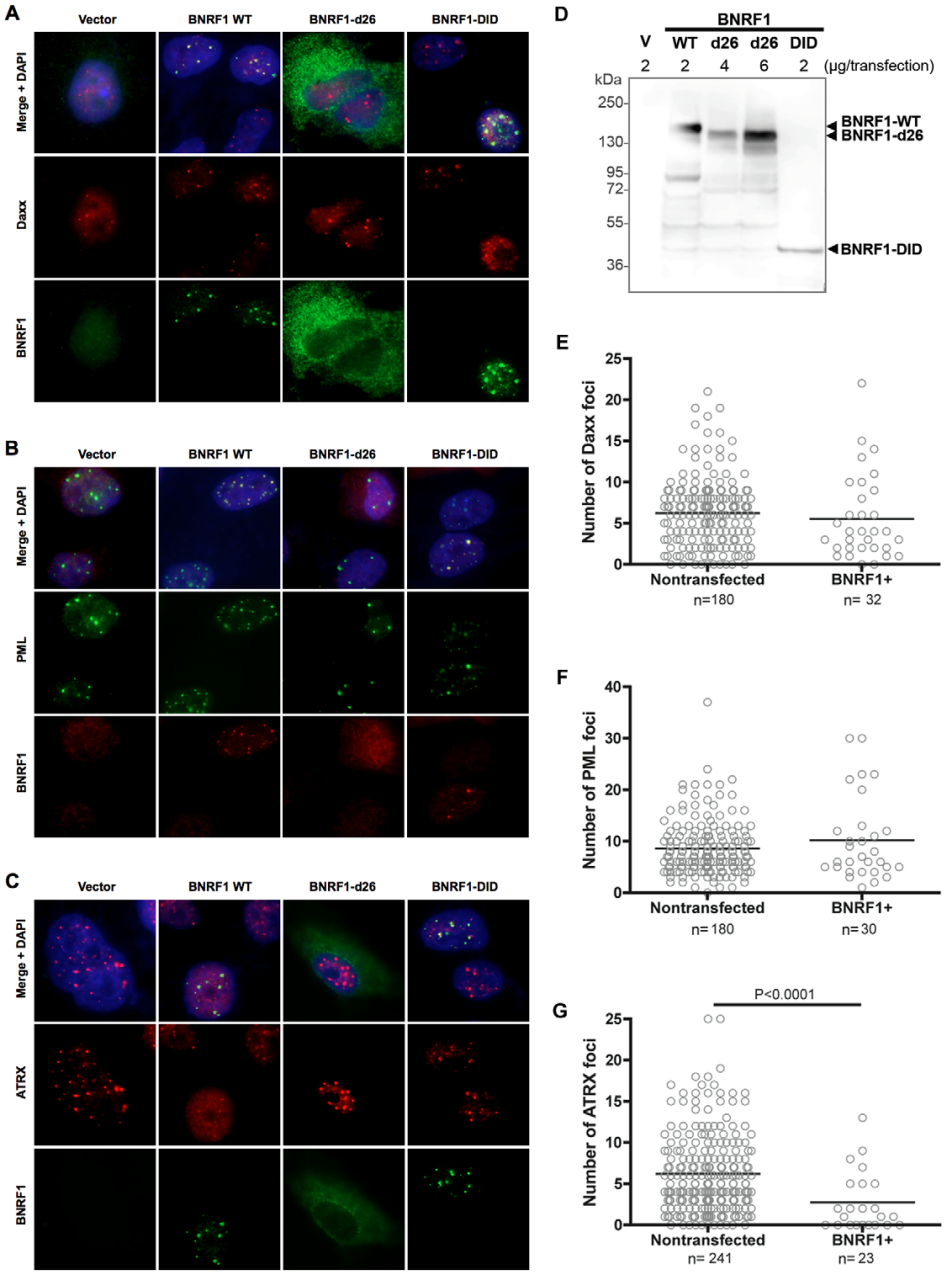
BNRF1 co-localizes with Daxx at PML-NBs and disperses ATRX from PML-NBs. Hep2 cells were transfected with either FLAG empty vector, WT BNRF1, or the deletion constructs d26 and DID. Cells were fixed 2 days post transfection and co-stained with anti-FLAG, and DAPI, and either anti-Daxx (A), anti-PML (B), or anti-ATRX (C) antibodies. Yellow regions in the merged panels denote co-localization of red and green signals. Remaining un-fixed, transfected Hep2 cells were subject to cell lysate Western blot analysis to confirm transfection efficiency and expression levels of the BNRF1 constructs (D). The number of nuclear bodies per cell nucleus was quantified by computational analysis of immunofluorescent microscopy images. A total of ten 40x magnification microscopy images of random fields were analyzed for each foci count. Foci counts of either Daxx (E), PML (F), or ATRX (G) were grouped into BNRF1 non-expressing and expressing sets. Bars on scatter plots denote the average foci per cell.

Daxx is a prominent component of PML nuclear bodies [47], and Daxx localization at these nuclear bodies are disrupted by viral proteins of both HSV-1 and HCMV [24], [34]. Thus, it is important to investigate the sub-cellular location of BNRF1-Daxx interaction, and check if BNRF1 disrupts Daxx localization to the nuclear bodies. For immunofluorescence (IF) microscopy studies, we selected Hep2 carcinoma cell lines because of their larger size and prominent PML nuclear bodies, and their common use in many previous studies with herpesvirus protein interactions with PML-NBs. Hep2 cells were transiently transfected with empty FLAG vector (V) or BNRF1 constructs WT, d26, or DID. Cells were then fixed two days post transfection and subject to IF staining. We found that WT BNRF1 partially co-localized with nuclear foci containing Daxx (Fig. 4A, S1A and Table S1), PML (Fig. 4B, S1B and Table S1), and Sp100 (Fig. S1D), suggesting that BNRF1 interacts with Daxx at the PML nuclear bodies. We also noticed that DID itself is sufficient for localizing to PML nuclear bodies, while the d26 deletion mutant showed a weak dispersed pattern in the cell (Fig. 4A and B, S1 and Table S1). The co-localizations were also confirmed by line scan analysis, where the BNRF1 WT and DID intensity peaks overlap with Daxx and PML peaks (Fig. S2). To ensure that the diffuse pattern of the d26 mutant is not due to deficient protein expression, the same set of transfected cells as used for IF were also assayed by Western blot for total expression levels of BNRF1 proteins (Fig. 4D). We found that d26 protein was expressed at levels similar to that of WT, despite its diffuse staining in IF studies, confirming its protein expression in the cells used in our microscopy study. These findings indicate that the interaction with Daxx is necessary and sufficient for BNRF1 to localize to the nuclear bodies.

To understand the BNRF1 disruption of Daxx-ATRX complex in a sub-cellular spatial context, we also examined ATRX by IF in BNRF1-transfected Hep2 cells (Fig. 4C and S1C). Again, we found ATRX foci co-localizing with WT BNRF1 and DID but not d26, which is also confirmed by line scan analysis (Fig. S2C). However, we also found a substantial reduction in ATRX foci intensity when cells were transfected with WT BNRF1, but no apparent reduction when transfected with d26 or DID-mutants (Fig. 4C). The failure of DID to disperse ATRX is consistent with its only partial disruption of ATRX from Daxx IP (Fig. 3C). Quantification of Daxx (Fig. 4E), PML (Fig. 4F), and ATRX (Fig. 4G) nuclear foci in BNRF1-expressing cells compared to non-expressing cells revealed that BNRF1-expressing cells contain a significantly lower (p<0.0001) average number of ATRX nuclear foci than non-expressing cells (Fig. 4G). In contrast, we found no significant difference in the number of Daxx (Fig. 4E) and PML nuclear foci (Fig. 4F) in BNRF1 transfected cells. Taken together, these results suggest that BNRF1 not only disrupts the Daxx-ATRX complex, but also actively disperses ATRX away from nuclear bodies.

**Figure 5:**
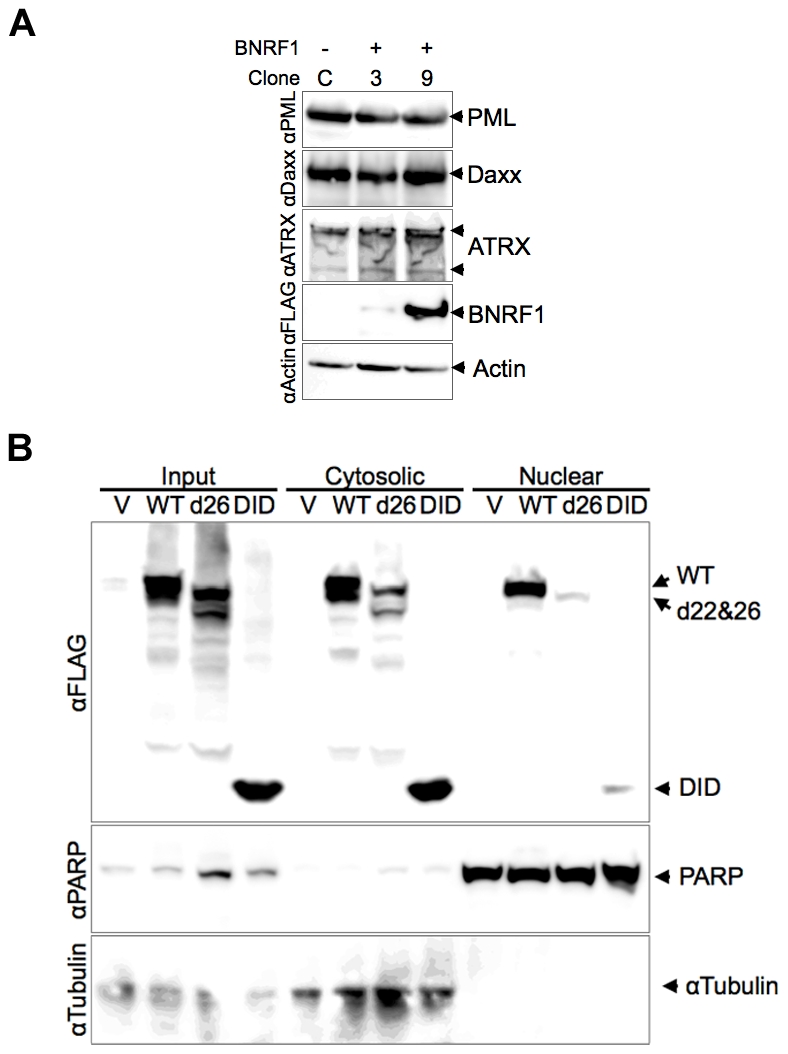
Stability and subcellular fractionation of BNRF1 proteins. (A) FLAG vector (clone C) or FLAG-BNRF1 (clones 3 and 9) stably transfected 293T cells were lysed and analyzed by Western blot. Blots were probed with antibodies to PML, Daxx, ATRX, FLAG (BNRF1), or Actin, as indicated to the right. (B) 293T cells transfected with FLAG vector (V), WT-BNRF1 (WT), d26, or DID mutant for 48 hrs were prepared as total cell extracts (input), cytoplasmic or nuclear fractions. Transfected cells fractions were assayed by Western blot with antibody to FLAG (BNRF1), PARP1 (nuclear marker) or αTubulin (cytoplasmic marker).

HSV-1 ICP0 and HCMV pp71 each induce the degradation of PML and Daxx proteins respectively, yet we did not observe any evidence of this with BNRF1 in our microscopy studies. To investigate the potential degradation of PML, Daxx and ATRX proteins by BNRF1, we examined the stability of these proteins in BNRF1 stably transfected cells (Fig. 5A). 293T cells stably transfected with control vector (clone C) or WT BNRF1 (stable transfection clones 3 and 9) were lysed and subject to Western blot analysis. We found no evidence of degradation or gross post-translational modification of PML, Daxx, nor ATRX in BNRF1-expressing cell lines. We also analyzed the protein stability of PML and Daxx in Hep2 cells transiently transfected with control vector, WT BNRF1 or d26, and again found no evidence of BNRF1-induced protein degradation (Fig. S3). This suggests that BNRF1 does not mimic the protein degradation function of HCMV pp71 or HSV-1 ICP0, but rather, disrupts Daxx-ATRX interactions through alternative mechanisms.

The diffuse distribution of the BNRF1-d26 mutant raised the question of whether the Daxx interaction domain of BNRF1 correlated with nuclear localization. To test this, we utilized biochemical fractionation methods to isolate nuclear and cytoplasmic proteins from 293T cells (Fig. 5B). We found that WT BNRF1 localized to both cytoplasmic (∼60%) and nuclear (40%) fractions. The d26 mutant, which is deficient in both Daxx interaction and nuclear bodies localization, was expressed at lower amounts yet showed a cytoplasmic to nuclear distribution similar to WT (Fig. 5B). This is consistent with d26 having a weak diffuse nuclear and cytoplasmic staining in IF (Fig. 4). Meanwhile, the DID mutant, which binds Daxx and co-localizes with PML nuclear bodies in the nucleus, was isolated at low, yet detectable levels in the nucleus; although substantially more was recovered in the cytoplasmic fraction. The efficiency of the fractionation was confirmed by the presence of PARP1 exclusively in the nuclear fractions, and α-tubulin exclusively in the cytoplasm. These findings suggest that BNRF1 can localize to both cytoplasmic and nuclear compartments, and that the Daxx interaction domain might contribute partially to the nuclear entry or stability of BNRF1.

### The Daxx interaction domain is required for BNRF1 to support EBV primary infection

**Figure 6:**
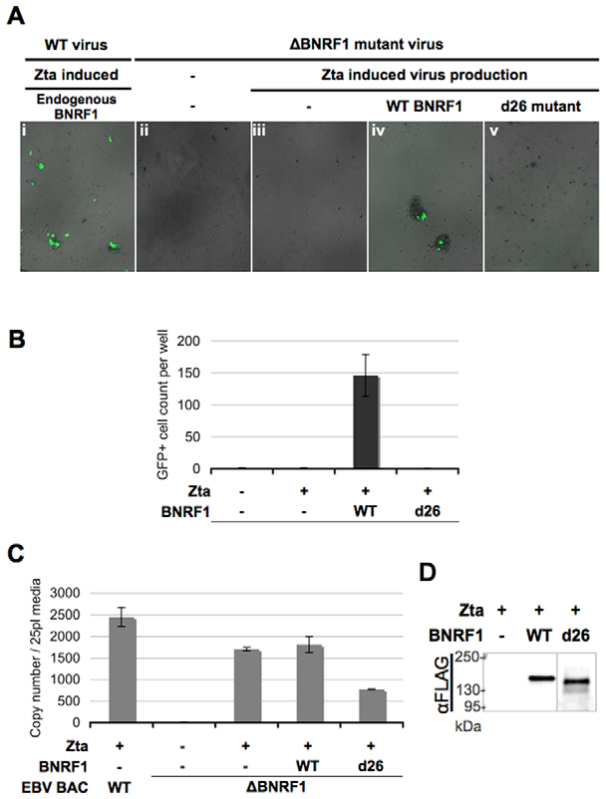
△BNRF1 mutant virus infection can be rescued by ectopic complementation with wild type, but not Daxx-interaction-deficient BNRF1. Primary B cells were infected with equal volumes of GFP expressing virus, either produced from wild type bacmids or △BNRF1 mutant virus complemented with empty FLAG vector, wild type, or the d26 Daxx-interaction-deficient mutant BNRF1. Infected cells would express the GFP carried in the virus. Infection rate as shown in fluorescent microscopy photos (A), or a manual count of the average number of GFP-positive cells per well (B). Aliquots of the virus used for infection were isolated for measurement of viral titers by real-time PCR analysis of the number of viral OriLyt DNA copy numbers (C). Viral particles reconstituted with FLAG vector, FLAG-tagged WT-BNRF1 or d26 mutant were concentrated and analyzed by Western blot with antibody to FLAG (D).

A previous study using an EBV bacmid with a BNRF1-knockout demonstrated that BNRF1-mutant virions can be generated from producer 293 cells and can enter the cytosol of infected B-cells; yet mutant virus failed to express one of the first expressed latent genes, EBNA2, upon primary infection of B cells, and were incapable of inducing B-cell proliferation [44]. To understand the role of BNRF1-Daxx interaction in primary infection, we took a complementation rescue approach with the BNRF1-mutant virus. 293 cells stably transfected with either wild type or BNRF1-knockout EBV bacmids were used for virus production (Fig. 6). As the EBV bacmids also encode GFP, cells infected with this bacmid-derived virus could be visualized by the presence of green fluorescence. To induce viral production, bacmid containing cells were co-transfected with the EBV transactivator Zta and BALF4. To complement for BNRF1 deletion, production cells were also transfected with either control vector, WT BNRF1 (WT), or the BNRF1 deletion mutant (d26) which fails to interact with Daxx. Three days after transfection, the media was collected and used to infect primary B cells isolated from human peripheral blood mononuclear cells (PBMCs). We detected high-levels of GFP positive proliferating B-cell clusters when infected with virus generated from wild type bacmid (Fig. 6Ai), but no GFP positive or clumped cells were detected when infected with no virus (Fig. 6Aii) or virus from un-complemented ΔBNRF1 bacmids (Fig. 6Aiii). However, when ΔBNRF1 virus was complimented with WT BNRF1 we were able to detect GFP positive cells and proliferating B-cell clusters (Fig. 6Aiv). Notably, ΔBNRF1 virus complimented with the d26 mutant BNRF1 failed to express GFP or induce B cell proliferation (Fig. 6Av), showing a similar defect as ΔBNRF1 virus with no complementation. Quantification of at least three independent infections confirmed that GFP positive and proliferating B-cells were detectable only when ΔBNRF1 bacmid virus was complemented with WT, but not with d26 mutant BNRF1 (Fig. 6B). To ensure the infections between each complemented virus were comparable, virus titer was quantified by real time PCR for virion DNA. The viral titers of either empty vector or WT BNRF1 complemented virus was found to be similar, while some reduction in virus titer was observed with d26 virus (Fig. 6C). We also tested by Western blotting for incorporation of FLAG-BNRF1 proteins in virions, and found that WT and d26 mutant BNRF1 proteins were both packaged into virions to similar per particle levels (Fig. 6D). These findings confirm that BNRF1 is required for primary infection of B-cells, and suggests that the Daxx interaction domain of BNRF1 is important for this function.

### BNRF1 promotes expression of the immediate early gene BZLF1

**Figure 7:**
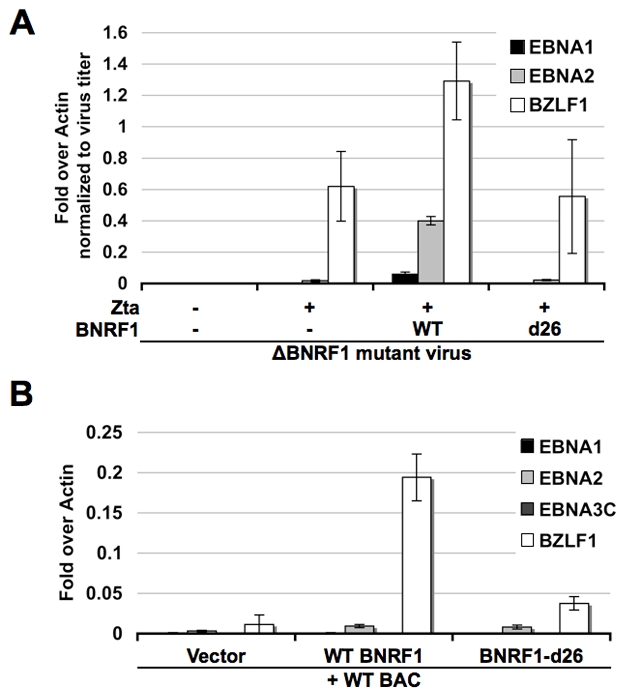
BNRF1 promotes viral gene expression. (A) Primary B cells were infected with virus produced from △BNRF1 mutant bacmids either complemented with empty vector, wild type BNRF1 or the d26 Daxx-interaction-deficient mutant BNRF1. Virus infected cells were subject to reverse transcription PCR assay of viral gene expression for EBNA1, EBNA2, or BZLF1. (B) 293HEK cells were co-transfected with wild type EBV genomes and either empty vector, WT BNRF1 or the d26 Daxx-interaction-deficient mutant BNRF1. Transfected cells were subject to reverse transcription PCR assay of viral gene expression for EBNA1, EBNA2, EBNA3C, or BZLF1.

Other herpesvirus tegument proteins that interact with Daxx and ATRX have been shown to function in the transcription activation of viral genes during primary infection [25]. To investigate the role of BNRF1 on viral gene transcription early after primary infection, we infected human B-lymphocytes purified from PBMCs with the ΔBNRF1 virus complemented with empty FLAG-vector, WT BNRF1 or d26 mutant BNRF1 (Fig. 7A). Viral gene expression in these newly infected cells was assayed at four days post infection using Reverse Transcription qPCR (RT-qPCR). We found that WT BNRF1 complementation induced an up-regulation of EBNA1, EBNA2 and BZLF1 mRNA expression compared with non-complemented virus or the d26-mutant complementation. Interestingly, background levels of BZLF1 expression were detectable in non-complemented and d26 mutant infections, suggesting that BNRF1 may only partly enhance BZLF1 expression, which can occur at low levels independently of BNRF1.

To investigate the potential mechanism of BNRF1 in viral gene regulation, we first tested the effect of BNRF1 on reporter plasmids using transient transfection assays, but found no consistent effect on candidate viral promoters (data not shown). We reasoned that reporter plasmids may lack essential BNRF1 target elements or chromatin assembly, and therefore assayed BNRF1 activity on EBV bacmid genomes after transfection into 293 cells (Fig. 7B). EBV bacmid DNA (Bac36) and either empty FLAG-vector, WT BNRF1, or the d26 mutant BNRF1 were co-transfected into 293 cells and assayed 3 days post transfection for viral gene expression using RT-qPCR. We found that WT BNRF1 promoted a robust expression of BZLF1 transcripts (∼20 fold), which was not observed in vector control or the d26 mutant (Fig. 7B). BNRF1 also increased EBNA2 mRNA (∼3 fold) relative to vector control, but this was not significantly increased relative to that of the d26 mutant. These studies suggest that BNRF1 can activate the expression of the EBV immediate early gene BZLF1 in the context of the viral genome, and in the absence of other virion-delivered tegument proteins.

### Daxx and ATRX restrict viral reactivation from latency

**Figure 8:**
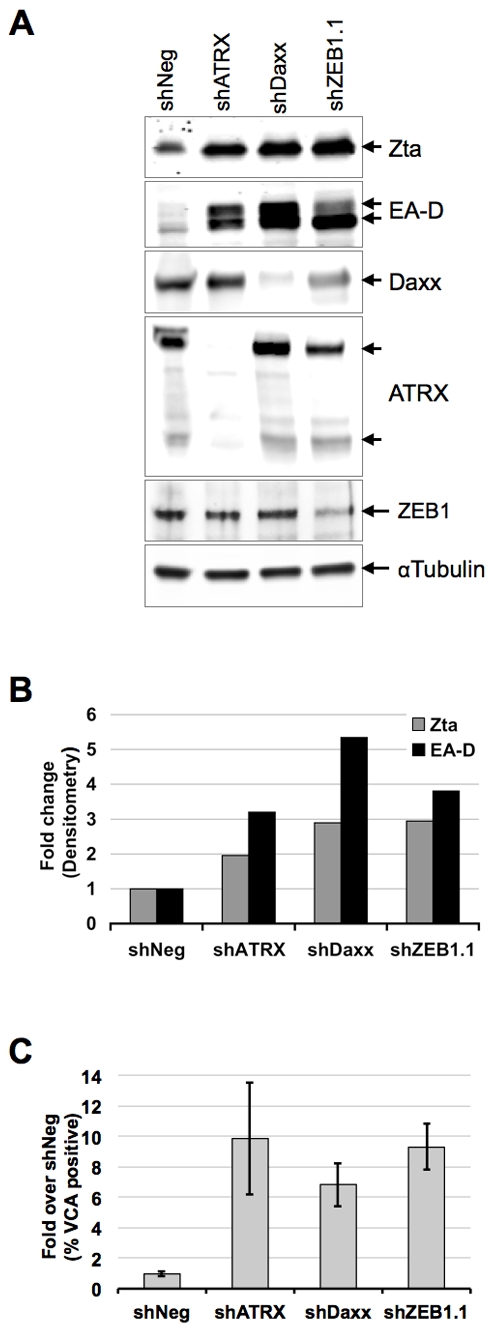
ATRX and Daxx depletion leads to reactivation of latent EBV. Mutu I cells were transduced with lentivirus shRNA with non-targeting sequence (shNeg), or targeting for ATRX (shATRX), Daxx (shDaxx) or positive control ZEB1 (shZEB1.1). Transduced cells were selected for puromycin resistance for 9 days and then assayed by Western blot with antibodies to Zta, EA-D, Daxx, ATRX, ZEB1, or αTubulin, as indicated and visualized by HRP (A) and densitometric scanning of Western blot band intensities (B). Three replicates of Mutu I transduced as described above were assayed by flow cytometry with antibody to EBV viral capsid antigen VCA and quantified as % VCA positive fold change relative to shNeg. Error bars denote the standard error among the independent experiments (C).

The previous experiments suggest that BNRF1 can function during tegument delivery in early infection, as well as after *de novo* synthesis, perhaps regulating the transition from latent to lytic infection. To explore the role of Daxx and ATRX in the context of EBV latent to lytic gene regulation, we test the effects of Daxx and ATRX knockdown on viral lytic gene expression in Mutu I cells, an EBV-latently infected Burkitt's lymphoma cell line (Fig. 8). Mutu I cells were transduced with puromycin resistant lentivirus carrying either non-targeting shRNA (shNeg), shRNA against Daxx (shDaxx), ATRX (shATRX), or ZEB1 (shZEB1.1) which acts as a positive control for reactivation. ZEB1 has been shown to repress Zta expression, and shRNA depletion of ZEB1 can reactivate lytic gene expression in several cell types [48], [49], [50]. Mutu I cells were harvested 9 days after shRNA transduction and selection, and then tested for viral reactivation by Western blot and FACS (Fig 8). Western blot analysis of whole cell lysates (Fig. 8A and B) revealed that knockdown of either Daxx or ATRX induced a reactivation of EBV early antigens, as shown by increased band intensities of both the immediate early gene Zta (2-fold) and the lytic early antigen EA-D (3-fold). These induction levels are comparable to that observed with the shZEB1.1 positive control. The efficiency of shRNA-mediated knockdown was confirmed by the loss of Daxx, ATRX and ZEB1 bands in the corresponding lanes (Fig. 8A). We also verified reactivation by flow cytometry quantification of the EBV viral capsid antigen VCA on cells from three independent shRNA-treatments (Fig. 8C), where we observed an approximately 6-to-10-fold induction by either Daxx or ATRX depletion. These findings indicate that the depletion of either Daxx or ATRX can promote viral lytic gene expression from latently infected B-cells, and suggest that BNRF1 disruption of the Daxx-ATRX complex contributes to viral gene control during early infection and reactivation.

## Discussion

The specific class of antiviral defense dubbed the intrinsic immunity [51], [52] plays a broad and general role in restricting viral infection. PML-NBs and its associated proteins such as PML, Sp100, Daxx and ATRX, have been extensively studied as cellular defenses against herpesviruses, specifically with the alphaherpesvirus HSV-1 and betaherpesvirus HCMV. Upon the early stages of infection right after cell entry, HSV-1 and HCMV utilize viral proteins that effectively disrupt the structure and disable the function of the PML-NBs in restricting viral gene expression and replication. However, the gammaherpesvirus EBV has been relatively less studied in terms of how it counteracts these cellular resistances upon primary infection or reactivation. We show here that the major tegument protein of EBV, BNRF1, interacts with Daxx (Figs. 1–3, S1–2) and disrupts its ability to form a complex with ATRX or recruit ATRX to PML-NBs (Figs. 3–4, S1–2). Moreover, we show that BNRF1 functionally promotes viral early gene expression with a preference for the activation of the immediate early gene BZLF1, and to a lesser extent the latent activator EBNA2 (Figs. 6–7). These findings indicate that EBV, like its relatives HSV1 and HCMV, encodes a viral tegument protein that targets PML-NB components to promote viral gene expression.

Daxx is a prominent PML-NB component, but is also associated with a diverse, yet non-mutually exclusive variety of cellular functions, including the regulation of apoptosis, chromatin remodeling, gene repression, and antiviral resistance [47], [53]. Daxx is a primary target of the HCMV pp71 protein, which both binds and induces the degradation of Daxx [30]. Like HCMV pp71, BNRF1 binds Daxx and prevents the Daxx-interaction partner, ATRX, from associating with Daxx and localizing to PML-NBs. BNRF1 and pp71 are both tegument proteins, whose pre-made nature likely provides them with a temporal advantage to disarm cellular repression machinery without the prior need of viral gene transcription. However, unlike HCMV pp71, BNRF1 does not induce Daxx degradation, which remains prominently associated with PML-NBs when BNRF1 is expressed (Figs 2–5). BNRF1 and pp71 share no obvious amino acid sequence similarity, and the Daxx interaction domains of these two proteins vary significantly in amino acid composition and size of the interaction domains. These findings suggest that BNRF1 is a functional homologue of pp71, but utilizes a distinct mechanism for the dissociation of ATRX from PML-NBs.

Herpesvirus tegument proteins have been implicated in the determination of viral lytic or latent gene expression programs. Restriction of tegument protein entry into the nucleus, as has been shown for HCMV pp71 and HSV VP16, correlates with the establishment of latency [24], suggesting that tegument proteins may play a critical role in determining lytic or latent gene expression programs. Interestingly, we found that the Daxx-interaction deficient BNRF1 mutant d26, which fails to interact with Daxx, showed a weak diffuse subcellular distribution instead of the punctate nuclear dots of WT BNRF1 (Fig. 4 and S1). Similarly, biochemical fractionation studies (Fig. 5B) suggest that while BNRF1 can localize to both the cytoplasm and nucleus, it may require the Daxx-interaction domain to efficiently accumulate in the nucleus. Potentially related is the observation that HCMV pp71 translocation to PML-NBs is also dependent on its interaction with Daxx [54]. Selective cytoplasmic retention of several herpesvirus tegument proteins, including pp71 and VP16, may play a critical role in determining lytic or latent gene expression programs [55]. We suspect that BNRF1 might be subject to similar regulation through its PML-NB localization.

Daxx and ATRX are known to play a global role in the control of cellular and viral gene expression and chromosomal structure. Daxx itself has been shown to associate with HDACs and to function as a global repressor of transcription [20], [21]. The Daxx-ATRX complex has *in vitro* chromatin remodeling activities [26] and can function as a histone H3.3 chaperone [56]. Recent studies suggest also that ATRX interacts with G-rich repeat chromatin regions [57], and in collaboration with Daxx load histone variant H3.3 onto pericentromeric and telomeric chromatin [56], [58], [59]. H3.3 is generally associated with open chromatin and active transcription when loaded by the histone chaperone HIRA [60]. However, the Daxx-ATRX complex loaded H3.3 has been found to facilitate transcription from pericentromeric regions [58] but repress transcription from telomeric regions [59]. Interestingly, HIRA-loaded H3.3 can facilitate the lytic replication of HSV-1 during the early steps of infection [61]. Furthermore, the Daxx-degrading pp71 blocks the establishment of heterochromatin on the HCMV Major Immediate Early Promoter (MIEP) region [25]. These findings underscore the importance of host chromatin regulatory mechanisms in the control of herpesvirus infection. We suspect that the viral gene activation function of BNRF1 (Fig. 7) is likely to be mediated by chromatin-dependent processes since we failed to observe consistent transcription activation when assayed in transient plasmid-based reporter assays using EBV promoters for BZLF1 (Zp) or EBNA2 (Cp or Wp) (data not shown). We propose that BNRF1 stimulates EBV early gene activity through de-repression of the Daxx-ATRX mediated chromatin repression mechanism, perhaps similar to that of pp71 de-repression of the HCMV MIEP locus. However, the precise molecular mechanism through which BNRF1 activates early gene transcription through the disruption of ATRX-Daxx interaction remains to be investigated.

While not explored yet, it is also not known if the FGARAT enzyme-homology domain of BNRF1 has any function in the context of supporting viral infection. This enzyme homology is conserved among all gammaherpesvirus orthologues of BNRF1, including the KSHV and MHV68 ORF75 family members. Despite significant sequence similarity with BNRF1, KSHV and MHV68 ORF75 proteins do not appear to interact with Daxx (data not shown). However, MHV68 ORF75c targets PML-NBs through the degradation of PML [45], [46], an activity that we did not observe with BNRF1. Thus, while these tegument family members share the FGARAT homology regions, and may similarly target components of the PML-NBs, they appear to target different proteins and utilize distinct mechanisms. It is also important to note that the disruption of ATRX by BNRF1 was partially dependent on the FGARAT domain, since the DID alone, which binds Daxx efficiently, only partially disrupt ATRX binding in IP assays (Figs 2 and 3) while not causing any significant ATRX dispersion from PML-NBs in IF assays (Fig. 4). Also, deletions within the FGARAT domain (d3 and d4) resulted in a mutant BNRF1 that co-precipitated with ATRX, creating a gain of function not seen with WT BNRF1. All of this suggests that the FGARAT domain may play a regulatory role in BNRF1 interactions with Daxx and ATRX.

In conclusion, our data demonstrates a novel example of herpesvirus tegument protein interacting with components of the cellular antiviral resistance. BNRF1 interaction with Daxx may provide several functions, including the establishment of a chromatin structure conducive to viral early gene activity. Our findings demonstrate that EBV, like other herpesviruses, confront the PML-NB associated intrinsic defenses through a viral factor that is available and active upon the early stages of infection, and shed light into the critical control mechanisms that govern the early events of EBV infection before the establishment of latency.

## Materials and Methods

### Ethics statement

Human B-lymphocytes were obtained from the Wistar Institute phlebotomy lab. All samples were from anonymous adult donors and approved by the Wistar Institute Institutional Review Board. Written informed consent was provided by study participants.

### Cells

Hep2 and 293T cells were grown in Dulbecco's modified Eagle medium (DMEM) supplemented with 10% fetal bovine serum (FBS), 20 mM GlutaMAX (Gibco), 100 U/ml penicillin and 100 µl/ml streptomycin. 293HEK cells were grown in minimum essential medium Eagle (MEM), supplemented with 10% FBS and 20 mM GlutaMAX (Gibco). DG75 and Mutu I cells are EBV negative and positive (respectively) Burkitt's lymphoma cell lines, grown in RPMI 1640 medium supplemented with 10% FBS, 100 U/ml penicillin and 100 µl/ml streptomycin. Peripheral blood mononuclear cells (PBMCs) were isolated from fresh donated human blood by density gradient centrifugation with Ficoll-Paque Plus purchased from GE healthcare. Primary B cells were then isolated from PBMCs using Dynabeads Untouched Human B Cell isolation kit (Invitrogen). All cells were grown in a 5% CO_2_ incubator at 37°C. Stable 293 cell lines expressing FLAG-BNRF1 (clone 3 and clone 9) and empty FLAG vector (clone C) were grown in DMEM as described for 293T cells above, supplemented with 2.5 µg/ml Puromycin for selection.

### Viruses bacmids and virus production cells

Viruses were produced using chloramphenicol and hygromycin resistant bacmids containing the EBV genome and the gene coding for green fluorescence protein (GFP). 293/EBV-wt cells (a gift from H. J. Delecluse) are 293HEK cells stably transfected with the wild type EBV bacmid [62]. 293/△BNRF1 cells (a gift from H. J. Delecluse) are 293HEK cells stably transfected with an EBV bacmid with the BNRF1 gene deleted [44]. 293/EBV-wt and 293/△BNRF1 cells were grown in RPMI 1640 medium supplemented with 10% FBS and 100 µg/ml hygromycin.

### Enzymes and antibodies

All restriction enzymes, T4 DNA ligase and associated buffers were purchased from New England Biolabs. Monoclonal mouse anti-FLAG antibody (F1804), Polyclonal rabbit anti-FLAG anibody (F7425), Polyclonal rabbit anti-Daxx antibody (F7810), Monoclonal Anti-α-Tubulin antibody (T5168), Monoclonal mouse anti-β-Actin-Peroxidase antibody (A3854), and Anti-mouse IgG R-Phycoerythrin (PE) conjugated antibody (P8547) were purchased from Sigma-Aldrich. Monoclonal mice anti-PML (PG-M3, sc-966), and polyclonal rabbit anti-ATRX (H-300, sc-15408), and polyclonal rabbit anti-ZEB1 (sc25388) were purchased from Santa Cruz Biotechnology. Polyclonal rabbit anti-PARP1 antibody (ALX-210-895-R100) was purchased from Enzo Life Sciences. Mouse anti-EA-D antibody was purchased from Millipore. Anti-EBV-VCA (0231) antibody was purchased from Pierce Thermo Scientific.

### Construction of BNRF1 expression plasmids and truncations

BNRF1 was cloned into the HindIII-SalI sites of the p3xFLAG-Myc-CMV-24 Expression Vector (Sigma-Aldrich), using the PCR primers: gcgaagcttgaagagaggggcagggaaacgcaa and gcggtcgactcactcggaggggcgaccgtgcctg. BNRF1 deletion mutants were generated as follows. PCR Primers (Table S2) were designed so that the front and rear halves of the DNA oligo each binds the 5′ or 3′ regions flanking the targeted deletion site on the BNRF1 template. PCR reactions were setup using iProof High-Fidelity DNA polymerase 2x master mix (Bio-Rad), with primers at 1 µM concentration, and the FLAG-BNRF1 expression plasmid as the template at a concentration of 50 ng DNA in a 25 µl reaction setup. PCR was done with a Bio-Rad C1000 thermal cycler, thermal cycles setup according to DNA polymerase mix manufacturer suggested conditions. To clear out the wild type BNRF1 template, 15 µl of the PCR product were treated with 30 U DpnI (New England Biolabs) in a 20 µl reaction for 2 hours to over night at 37°C. 2 µl of DpnI-treated DNA were then transformed into 50 µl of Library Efficiency DH5α competent cells (Invitrogen). Colonies were screened for the deletion by enzyme digestion analysis of miniprep DNA, and then confirmed by DNA sequencing of the expected deletion site.

### Immunoprecipitation

BNRF1 expression plasmids were transfected using Lipofectamine 2000 (Invitrogen) according to manufacturer instructions. Cells were harvested 2 days post transfection by washing cells off the plate with PBS. Harvested cells were further washed 3 times with cold PBS, and then subject to lysis with freshly prepared NET lysis buffer (50 mM Tris-HCl pH 7.5, 150 mM NaCl, 5 mM EDTA, 0.5% NP-40, and 0.1% mammalian protease inhibitor cocktail mix (P8340, Sigma-Aldrich), at 1 ml NET per IP pull-down. Cell lysates were homogenized by doing 10 strokes in a Dounce homogenizer. 60 µl of each lysate were isolated after this step as input control. The remaining lysates were incubated at 4°C rotating for 30 mins to fully solubilize proteins. Lysates were then spun at 13000 rpm 5 mins to remove insoluble cell debris, then antibodies were added (5 µl of each antibody per IP) to the cleared lysates, and left rotating over night. 100 µl of 50% slurry of Protein A sepharose beads (GE healthcare) in NET buffer was added to each IP with rotating at 4°C for 2–3 hours, then washed three times with NET for 10 mins (rotating at 4°C) per wash. Pulled down proteins were released by adding 50 µl 2x Laemmli buffer (100 mM Tris-Cl pH 6.8, 4% SDS, 0.2% Bromophenol Blue, 20% Glycerol), and boiling for 10 mins at 100°C. The resulting samples (excluding beads) were then loaded directly into protein gels and subject to Western blot analysis.

For mass spectrometry identification of BNRF1 associated proteins, FLAG-BNRF1 expressing and FLAG-vector control stable cell lines were generated as mentioned above. Nuclear extracts from 5×10^7^ cells were subject to immunopurification with anti-FLAG Sepharose beads (A2220, Sigma-Aldrich) followed extensive washing with NET buffer, and FLAG peptide elution. Eluted protein was subject to precipitation with 10% trichloroacetic acid (TCA) followed by SDS-PAGE and colloidal blue staining. Sections of the gel with enriched polypeptides were subject to LC/MS/MS at the Wistar Proteomics Facility.

### Immunofluorescence microscopy

Hep2 cells were transfected with BNRF1 expression plasmids using Lipofectamine 2000 (Invitrogen) according to the manufacturer's instructions, transfected cells were then reseeded at 2.7×10^4^ cells/well in 24 well plates containing microscope coverslips 5 hours post transfection. 2 days post transfection, coverslips with cells attached were harvested, fixed with 1% paraformaldehyde at room temperature for 15 mins, then permeablized with 0.3% Triton-X 100. Coverslips were then stained with the first antibodies over night at 4°C. First antibody dilutions used were as follows: mouse anti-FLAG at 1∶20000, rabbit anti-Daxx at 1∶5000, rabbit anti-FLAG at 1∶5000, mouse anti-PML at 1∶250, rabbit anti-ATRX at 1∶250, all antibodies diluted in PBS. Second antibody stainings were carried out for 1 hour at room temperature with the red-fluorescent Alexafluor594 goat anti-rabbit antibody and green Alexafluor488 goat anti-mouse antibody (both from Invitrogen) each at 1/800 dilution in PBS. Coverslips were washed twice in PBS for 5 mins between each of the above treatments. Cell nuclei were stained briefly with DAPI (diluted to a final concentration of 0.167 µg/ml in PBS) for 2 mins, then washed with PBS, 70% EtOH, then 100% EtOH to wash out residual salts. Coverslips were air-dried briefly, and then mounted onto microscope slides with Vectasheld mounting media (Vector Laboratories). Mounted slides were examined under a Nikon E600 upright microscope with a 100x oil objective. Photos for nuclear body quantification were took using a 40x objective to maximize the number of cells in each photo while retaining a clear view of PML bodies.

### Nuclear body quantification

Microscopy photos were analyzed using ImagePro Plus 6.2 software (Media Cybernetics). Photos were pre-processed by subtracting out the background intensity using the operation function (with a value of −30), and passing through a flatten filter (a value of 10). A morphological ‘top hat’ filter was then applied to emphasize points or grains brighter then the background. The number of nuclear bodies in each cell nucleus was counted by quantifying the object numbers after applying the signal intensity threshold/segmentation tool to select the nuclear bodies as objects. Cell boundaries were defined by the outline from DAPI channel photos of the same field, while omitting all cells on the border of the image border. Resulting quantification numbers were then analyzed using Prism 4 software (Graph Pad Software), statistical analysis did by Mann-Whitney U non-parametric, unpaired t test.

### Subcellular fractionation assay

293T cells were transfected in 10 cm plates with 2 µg expression plasmids of either empty FLAG vector, WT-BNRF1, BNRF1-DID, or 6 µg of BNRF1-d26. Transfection was carried out using 10 µl Lipofectamine 2000 (Invitrogen) per transfection, following manufacturer instructions. Cells were harvested 24 hrs post transfection. 1/6 of cells isolated as input control. The rest of the cell pellets were fractionated with the Fermentas ProteoJET Cytoplasmic and Nuclear Protein Extraction Kit (K0311). The resulting cytoplasmic and nuclear fractions, along with the input samples, were analyzed by Western blot.

### BNRF1 complementation virus infection assay

To induce lytic virus production, 293/EBV-wt and 293/△BNRF1 cells were transfected in 10 cm plates with expression plasmids of 1.75 µg BALF4, 3.25 µg BZLF1 or cDNA3 empty vector, and 3 µg of either empty FLAG vector or 3 µg BNRF1 or 7.5 µg BNRF1-d26. Transfection was carried out using 15 µl Lipofectamine 2000 (Invitrogen) per transfection, following manufacturer instructions. The media of virus production cells were harvested 3 days post transfection, filtered through 0.45 µm filters, and added directly to freshly isolated primary B cells. B cells in virus containing media were centrifuged for 1200 rpm 90 mins at 25°C to enhance infection.

For measuring infection by GFP levels, infected B cells were treated with 1 mM Sodium Butyrate and 20 ng/ml TPA 3 days post transfection to enhance GFP expression, and the number of GFP positive cells in each well were counted manually under a Nikon TE2000 microscope using a 20x objective.

For measuring virus gene expression in infected B cells, cells were collected 4 days post transfection, and total RNA was purified using Trizol (Invitrogen). The resulting RNA was then subject to DNase 1 treatment at 2 U/50 µl, 1 hour at 37°C, then DNase was heat inactivated by adding a final concentration of 5 mM EDTA and incubated at 70°C for 10 mins. cDNA was synthesized using the Super Script III first strand synthesis system reverse-transcription kit (Invitrogen). The resulting cDNA was then subject to real time PCR analysis by ΔCt method and normalized to viral titers, measured as described below. Real time PCR primers used are listed in Table S3.

To measure the amount of complemented BNRF1 protein that were packaged into virions, △BNRF1 virions complemented with WT-BNRF1, BNRF1-d26, or un-complemented, were produced as mentioned above. 100 µl of the harvested and filtered virus-containing media were isolated for viral titer measurement as described below. The rest of the virus-containing media were concentrated by loading the media above a 5 ml layer of 22.5% sucrose in PBS, then centrifuged at 27000 rpm (∼100,000 g) 4°C for 1 hour in a SureSpin 630 Rotor (Thermo Scientific) with a Sorvall WX 100 Ultra ultracentrifuge. The resulting virus pellet was then resuspended in PBS, and analyzed by Western blot. Protein gel loading volumes were normalized according to viral titers to ensure equal amounts of virion protein in each well.

### Virus titer measure by real time PCR detection of DNA copy number

Viral DNA in media was extracted as described by C. Busse et al. [63]. Virus-containing media were treated with 5 U/50 µl of DNase I (New England Biolabs) for 1 hour at 37°C. DNase was then deactivated by adding EDTA to a final concentration of 5 mM, followed by 10 mins heat inactivation at 70°C. Samples were then mixed 1∶1 with 0.1 mg/ml of proteinase K in water, and incubated at 50°C for 1 hour, followed by 20 mins of heat inactivation at 75°C. The released viral DNA was measured by real time PCR analysis, using a serial dilution series of Namalwa cell lysate as the standard curve, which contain two copies of integrated EBV genome per Namalwa cell. EBV genomes were detected using primers specific to the OriLyt region: 5′- CGTCTTACTGCCCAGCCTACTC-3′ (OriLyt-fwd), 5′- AGTGGGAGGGCAGGAAATG-3′ (OriLyt-rev).

### Virus gene expression assay on bacmid/BNRF1 co-transfected cells

Wild type EBV genome bacmids were prepared from 2.5 mls overnight LB culture using the Bacmax DNA purification kit (Epicentre). 293HEK cells were seeded (2.3 million cells per plate) the previous day in 10 cm plates, and transfected with 1.5 µg freshly prepared bacmids along with 0.5 µg of either empty FLAG vector, BNRF1, or BNRF1-d26 mutant. Transfection was carried out using Effectene transfection reagents (Qiagen), following manufacturer instructions. Cells were harvested three days post transfection, total RNA was purified using Trizol (Invitrogen), and then subject to DNase 1 treatment at 2 U/50 µl, 1 hour at 37°C. DNase was heat inactivated by adding a final concentration of 5 mM EDTA and incubated at 70°C for 10 mins. cDNA was then synthesized using the Super Script III first strand synthesis system reverse-transcription kit (Invitrogen). The resulting cDNA was then subject to real time PCR analysis by ΔCt method. Real time PCR primers used are listed in Table S3.

### shRNA-mediated knockdown of genes

shNeg (pLKO-shNeg), shDaxx (pLKO-shDaxx-2) and shATRX (pLKO-shATRX90) constructs in lentivirus production plasmid backbones were generous gifts from Roger Everett. shNeg (sequence TTATCGCGCATATCACGCG) was designed to poorly target the E.coli DNA polymerase and extensively screened to ensure that it does not affect human nor viral transcripts. Use of shDaxx and shATRX was previously described else where [30], [31]. shZEB1.1 was obtained from the TRC library (Sigma, Inc), with targeting sequence GCAACAATACAAGAGGTTAAACTCGAGTTTAACCTCTTGTATTGTTGC). Mutu I cells were infected with lentiviruses carrying pLKO.1-puro vectors by spin-infection at 400 g for 45 minutes at room temperature. The pellets were resuspended in fresh medium and left growing overnight. The RPMI medium was replaced each day, with 2.5 ug/ml Puromycin added for selection for lentivirus transduced cells. The cells were collected after 9 days of puromycin selection, and subject to Flow cytometry quantification of EBV viral capsid antigen positive cells, and Western blot analysis.

## Supporting Information

Figure S1 **BNRF1 co-localizes with Daxx at PML-NBs and disperses ATRX from PML-NBs.** Hep2 cells were transfected with either FLAG empty vector, WT BNRF1, or the deletion constructs d26 (or d2 in panel D) and DID. Cells were fixed 2 days post transfection and co-stained with anti-FLAG, and DAPI, and either anti-Daxx (A), anti-PML (B), anti-ATRX (C), or anti-Sp100 (AB1380, Chemicon International, used at 1/800 dilution in PBS) antibodies. Yellow regions in the merged panels denote co-localization of red and green signals.
(TIF)

Figure S2 **Signal intensity profiles analysis of BNRF1 co-localization with PML nuclear bodies.** Color channel merged panels from Fig. 4 were subject to line scan signal intensity analysis as [64]. Signal intensity plots of red and green channels were plotted bellow each photo, where the x-axis runs from left to right along the yellow line drawn across several nuclear foci in each photo. Overlaps of BNRF1 signals with Daxx (A), PML (B), and ATRX (C) are analyzed.
(TIF)

Figure S3 **Time-course study of the effects of BNRF1 on PML and Daxx protein stability.** Hep2 cells were transfected with vector control, WT BNRF1, or BNRF1-d26 mutant expression vectors. Total cell lysates were analysed by Western blot at 0, 12, 24, or 48 hrs post-transfection. Western blots were probed with antibodies to PML, Daxx, FLAG (BNRF1), or Actin, as indicated to the right.
(TIF)

Table S1 **Percentage of BNRF1 foci overlap with Daxx or PML foci, as observed by IF microscopy.**
(XLS)

Table S2 **Primers used in site-directed mutagenesis of BNRF1 deletion constructs.**
(XLS)

Table S3 **Primers used for real time PCR detection of viral gene expression.**
(XLS)
