# Supplementary figures and images for: EBV Tegument Protein BNRF1 Disrupts DAXX-ATRX to Activate Viral Early Gene Transcription
Source: PLoS Pathog. 2011 Nov 10;7(11):e1002376. doi: 10.1371/journal.ppat.1002376 (PMC3213115; doi:10.1371/journal.ppat.1002376)

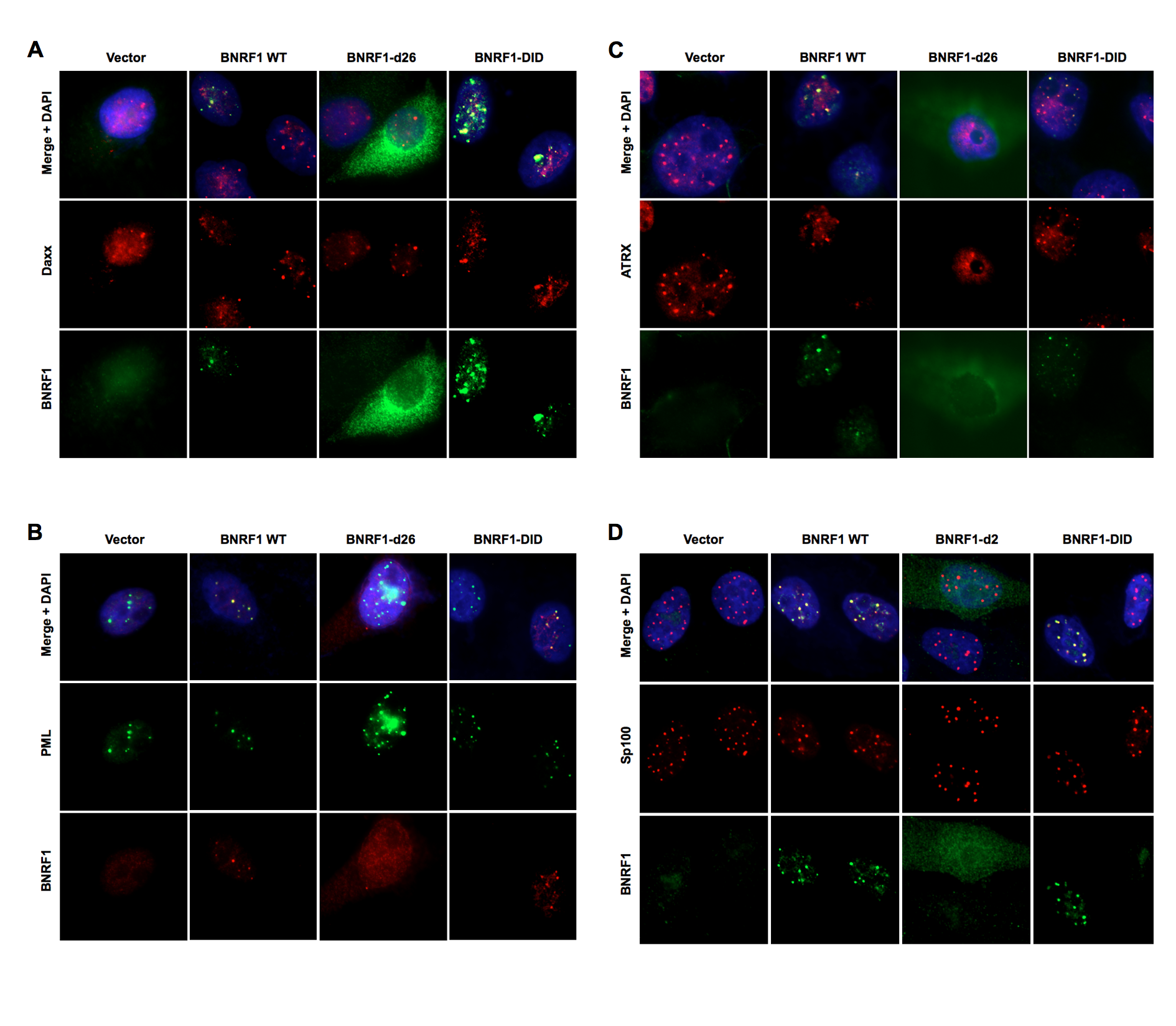

Supplement: Figure S1 — BNRF1 co-localizes with Daxx at PML-NBs and disperses ATRX from PML-NBs. Hep2 cells were transfected with either FLAG empty vector, WT BNRF1, or the deletion constructs d26 (or d2 in panel D) and DID. Cells were fixed 2 days post transfection and co-stained with anti-FLAG, and DAPI, and either anti-Daxx (A), anti-PML (B), anti-ATRX (C), or anti-Sp100 (AB1380, Chemicon International, used at 1/800 dilution in PBS) antibodies. Yellow regions in the merged panels denote co-localization of red and green signals. (TIF) [file ppat.1002376.s001.tif]

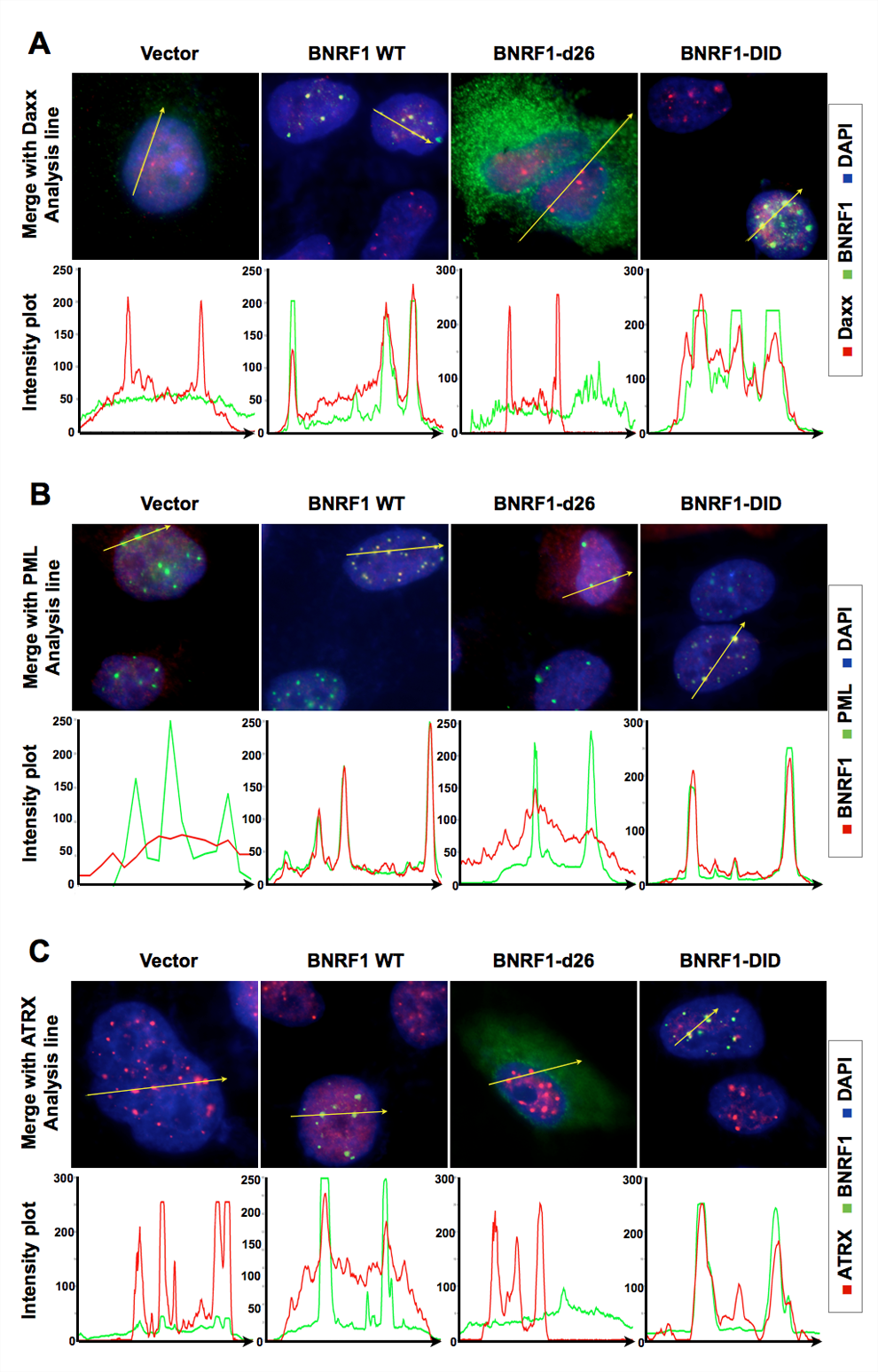

Supplement: Figure S2 — Signal intensity profiles analysis of BNRF1 co-localization with PML nuclear bodies. Color channel merged panels from Fig. 4 were subject to line scan signal intensity analysis as [64]. Signal intensity plots of red and green channels were plotted bellow each photo, where the x-axis runs from left to right along the yellow line drawn across several nuclear foci in each photo. Overlaps of BNRF1 signals with Daxx (A), PML (B), and ATRX (C) are analyzed. (TIF) [file ppat.1002376.s002.tif]

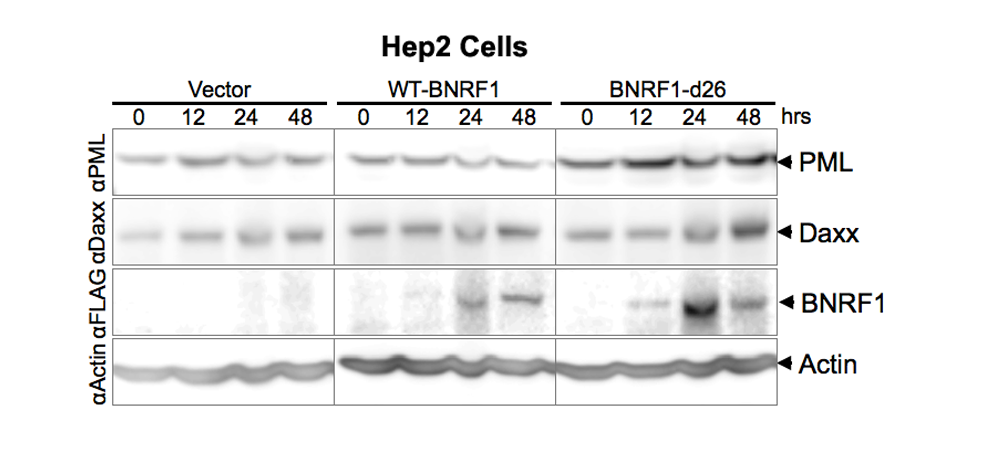

Supplement: Figure S3 — Time-course study of the effects of BNRF1 on PML and Daxx protein stability. Hep2 cells were transfected with vector control, WT BNRF1, or BNRF1-d26 mutant expression vectors. Total cell lysates were analysed by Western blot at 0, 12, 24, or 48 hrs post-transfection. Western blots were probed with antibodies to PML, Daxx, FLAG (BNRF1), or Actin, as indicated to the right. (TIF) [file ppat.1002376.s003.tif]
